# A retrospective cohort study: do patients with graves’ disease need to be euthyroid prior to surgery?

**DOI:** 10.1186/s40463-018-0281-z

**Published:** 2018-05-21

**Authors:** Abrar Al Jassim, Tim Wallace, Sarah Bouhabel, Agnieszka Majdan, Michael Hier, Veronique-Isabelle Forest, Richard Payne

**Affiliations:** 1 Department of Otolaryngology – Head and Neck surgery, Jewish General Hospital, McGill University, 3755 Côte-Sainte-Catherine Road, Montreal, Quebec, H3T 1E2 Canada; 2 Department of Otolaryngology – Head and Neck surgery, Cumberland Regional Health Care Center, Dalhousie University, Halifax, NS Canada; 3 Division of Endocrinology, Jewish General Hospital, McGill University, Montreal, Quebec, Canada

**Keywords:** Graves’ disease, Thyroid storm, Thyroidectomy, Hyperthyroid, Thyroid hormones, American Thyroid Association guidelines

## Abstract

**Background:**

The 2016 American Thyroid Association guidelines indicate that patients with Graves’ disease who undergo a thyroidectomy should be rendered euthyroid through the use of antithyroid drugs (ATD) prior to surgery to avoid complications such as a thyroid storm. At times, the use of ATDs can have limited efficacy and therefore some patients will inevitably remain biochemically hyperthyroid at the time of surgery.

The aim of this study is to assess if hyperthyroid patients undergoing a thyroidectomy are at an increased risk of developing a thyroid storm in comparison to euthyroid patients. Furthermore, this study seeks to establish a correlation between thyroid storm identified by the levels of thyroid hormones (T3 and T4) and the level of thyroid stimulating hormone (TSH).

**Methods:**

A retrospective cohort study was conducted at two Canadian centers, one in Montreal and the other in Nova Scotia. Sixty-seven patients undergoing thyroidectomy for Graves’ disease from January 2006 to December 2016 were evaluated.

**Results:**

The study comprised 67 participants with a mean age of 46 years (range16–78 years). A total of 78% of patients were on methimazole, 34% on beta-blockers, 27% on potassium iodine solution, 10% on propylthiouracil and 7% on steroids. At the time of surgery 21% were in an overt hyperthyroid state and 33% were in a subclinical hyperthyroid state. The average TSH level of 0.03 mIUL/L (range 0.01–0.23 mIUL/L). Sixteen percent of patients had a TSH level less than 0.01 mIUL/L. The average free T4 level was 29.58 pmol/L (range 11.5–95.2 pmol/L). The average total T3 level was 11.52 nmol/L (range 4.5–29.1 nmol/L) and free T3 level was 6.35 pmol/L (range 6.1–6.6 pmol/L). No patient developed thyroid storm.

**Conclusions:**

In our study, biochemically hyperthyroid patients undergoing thyroidectomy did not develop thyroid storm. Additional studies with larger sample sizes are needed to better understand the risk of thyroid storm in hyperthyroid patients.

## Background

Graves’ disease (GD) is an autoimmune disorder caused by the production of autoantibodies against the thyroid stimulating hormone receptor. This stimulates follicular cells to synthesize and secrete thyroid hormone, which in turn leads to hyperthyroidism [1]. The latter is defined by a low thyroid stimulating hormone (TSH) level and by an elevated serum triiodothyronine (T3) and thyroxine (T4) levels. GD is associated with diffuse goiter, ophthalmopathy, dermatopathy and a rare life threatening complication known as thyroid storm: a hypermetabolic state causing multiple organ dysfunction [2].

Currently, GD is managed by antithyroid drugs (ATD), radioiodine ablation (RAI) and surgery or a combination of thereof. In North America, RAI and ATDs are most commonly used methods of treatment. Surgery is indicated when radioiodine ablation and antithyroid medication are not successful, or in the cases of malignancy, planning of pregnancy within the next 6 months, mechanical compression due to a large goiter or severe ophthalmopathy [2]. In preparation for thyroid surgery, the American Thyroid Association guidelines recommend rendering the patient euthyroid and effectively ‘beta-blocked’ to decrease the risk of a potentially lethal thyrotoxic crisis [2]. This complication may be precipitated by the stress of surgery, anesthesia, or thyroid manipulation. Rendering the patient euthyroid requires 3 to 12 weeks of treatment with antithyroid drugs such as thionamides or propylthiouracil [1]. The use of antithyroids drugs is a lengthy process with limited efficacy and therefore some patients will inevitably remain biochemically hyperthyroid at the time of operation.

GD is associated with a higher serum T3 than serum T4 concentration and this ratio is used for outcome prediction in regards to response to therapy [3]. However, the current literature has no established cutoffs of serum T4 or T3 that differentiates uncomplicated thyrotoxicosis from crisis [4, 5]. Akamizu et al. [6], noted that in several cases of thyroid storm the patients had normal serum free T3 levels with increased serum free T4 levels and suppressed TSH levels. In more severe illness, some patients had low serum free T4 levels and TSH levels. In general, the mean serum free T4 and free T3 concentrations were not significantly different amongst patients in a hyperthyroid state compared to patients with thyroid storm [4]. Furthermore, some case studies identified patients with a thyroid storm that had normal serum T4 and excess serum T3 levels [7]. Therefore, it is difficult to safely predict the development of a thyroid storm in this specific patient population and establish acceptable cutoffs of thyroid hormone levels for patients undergoing surgery in a hyperthyroid state.

## Objective

The aim of this study is to assess if hyperthyroid patients undergoing a thyroidectomy are at an increased risk of developing a thyroid storm in comparison to euthyroid patients. Furthermore, this study seeks to establish a correlation between thyroid storm identified by the levels of thyroid hormones (T3 and T4) and the level of thyroid stimulating hormone (TSH).

## Methods

### Design

This is a retrospective cohort study.

### Ethics approval

Institutional ethics approval was obtained through our Ethics Board Committee prior to the study.

### Recruitment site

Patients were recruited from two Canadian centers, one in Montreal and the other in Nova Scotia. This included the Jewish General Hospital and the McGill University Health Centre, and Cumberland Regional Health Care Center affiliated with Dalhousie University.

### Patients’ selection

Patients were obtained from the practice of four surgeons, three in Montreal and one in Nova Scotia. The surgeons identified the patients who had the diagnosis of Graves’ disease and underwent thyroidectomy from January 2006 to December 2016. Graves’ disease diagnosis was confirmed by reviewing their TSH receptor antibody status, radioactive iodine uptake study or referral letter from endocrinologist. All patients with confirmed diagnosis of Graves’ disease and who underwent thyroidectomy were included in the study.

### Data recorded

The electronic medical records were reviewed and information was recorded in an electronic spreadsheet. Data collected included demographic information (age, gender, hospital number), relevant clinical symptoms associated with Graves’ disease (hypertension and ophthalmopathy), pre-operative medication use (methimazole, beta-blockers, propylthiouracil, potassium iodine solution and steroids), pre-operative thyroid hormone levels (TSH, T3 and T4), intra and postoperative occurrence of thyroid storm and type of surgical procedure, when available.

Patient thyroid hormone status (euthyroid, hypothyroid, subclinical hypothyroid, hyperthyroid and subclinical hyperthyroid) was also recorded. The cut off values used were based on the respective institution. In one center, the following ranges were used: TSH = 0.4–4.5mIUL/L, total T3 = 2.8–7.1 nmol/L, free T4 = 9–26 pmol/L. In the other center, the following ranges were used: TSH = 0.35–4.3mIUL/L, free T3 = 3.5–5.9 pmol/L, free T4 = 9-19 pmol/L. Hyperthyroid status was defined as low thyroid stimulating hormone (TSH) level and by an elevated serum triiodothyronine (T3) and thyroxine (T4) levels. Subclinical Hyperthyroidism was defined as low thyroid stimulating hormone (TSH) level and normal serum triiodothyronine (T3) and thyroxine (T4) levels.

### Data analysis

Statistic analysis was done using R software (version 3.3.2). The mean, median, range and 95% confidence interval was calculated for the TSH, total T3, free T3 and free T4 level for the euthyroid, subclinical hyperthyroid and overt hyperthyroid group. The 95% confidence interval was calculated for the occurrence of thyoid storm in hyperthyroid group.

**Table:** 

Characteristics	*n* (%)
**Age** mean (range)	46 (16–78)
**Sex**	
Male	10 (15)
Female	57 (85)
**Graves’ ophthalmopathy**	24 (36)
**Preoperative medications**	
Methimazole	52 (78)
Propylthiouracil	7 (10)
Potassium iodine	18 (27)
Steroids	5 (7)
Beta-Blockers	23 (34)
No medications	7 (10)

## Results

### Patients’ demographics

Between January 2006 to December 2016, 67 patients who underwent thyroidectomy as a means of treatment for their Graves’ Disease were evaluated; 50 patients were from Center 1 and 17 patients were from Center 2. 57 were female and 10 were male, with a mean age of 46 (range16–78).

### Patient’s pharmacological profile

A total of 78% of patients were on methimazole, 34% on beta-blockers, 27% on potassium iodine solution, 10% on propylthiouracil and 7% on steroids. Ten percent of patients were on no medications, 46% were on at least 1 drug and 20% were on more than 3 drugs.

### Patient’s thyroid hormone status

From the 67 patients, 22 patients were in a subclinical hyperthyroid state, 14 were in a hyperthyroid state and 11 patients had a TSH of less than 0.01 mIUL/L. 22 patients were in euthyroid state, 6 were in a hypothyroid state and 3 were in a subclinical hypothyroid state.

For all the patients in a hyperthyroid state, the average TSH level was 0.03 mIUL/L (range 0.01–0.23 mIUL/L) with 16% of patients having a TSH level less than 0.01 mIUL/L. The average total T3 level was 11.52 nmol/L (range 4.5–29.1 nmol/L). The average free T3 level was 6.35 pmol/L (range 6.1–6.6 pmol/L). The average free T4 level was 29.58 pmol/L (range 11.5–95.2 pmol/L).

**Table:** 

	Euthyroid	Hyperthyroid	Subclinical Hyperthyroid	TSH < 0.01
*N* (%)	22 (33)	14 (21)	22(33)	11(16)
TSH (mIU/L)				
Mean (Range)	2.7 (0.69–4.19)	0.03 (0.01–0.23)	0.095 (0.01–0.37)	0.01 (N/A)
Median	2.845	0.02	0.04	0.01
95% CI	2.2, 3.2	−0.0017, 0.064	0.047, 0.14	N/A
Total T3 (nmol/L)				
Mean (Range)	4.55 (3.9–5.3)	11.52 (4.5–29.10)	4.64 (2.34–7)	8 (3.7–14.2)
Median	4.6	9.8	4.8	6.9
95% CI	4.05, 5.06	4.89, 18.15	3.75, 5.54	4.2, 11.7
Free T3 (pmol/L)				
Mean (Range)	7.8 (4.2–18.1)	6.35 (6.1–6.6)	4.8 (N/A)	4.8 (N/A)
Median	4.45	6.35	4.8	4.8
95% CI	−3.12, 18.72	3.17, 9.52	N/A	N/A
Free T4 (pmol/L)				
Mean	13.92 (6.4–44.9)	29.58 (11.5–95.2)	13.08 (8.4–17.5)	20.4 (8.9–39.5)
Median	12	25.15	14.2	20.1
95% CI	8.8, 18.9	18.02, 41.15	11.79, 14.36	14.4, 26.4

### Complications

No patient developed thyroid storm; the 95% confidence interval is [0–0.08], this suggest up to 92% of patients undergoing surgery in a hyperthyroid state will not develop a thyroid storm.

## Discussion

The 2016 ATA guidelines recommend that if surgery is chosen as a treatment for Graves’ disease then the patient should be rendered euthyroid prior to the procedure with antithyroid drugs such as methimazole. Furthermore, potassium iodine preparations should be administered in the immediate preoperative period. This is to minimize complications and avoid a life threatening complication known as thyroid storm, which may be precipitated by the stress of surgery [2]. In our study, 78% of patients were on methimazole, 34% on beta-blockers, 27% on potassium iodine solution, 10% on propylthiouracil and 7% on steroids. At the time of surgery 21% of the patient remained biochemically hyperthyroid and no patient experienced a thyroid storm. The 95% confidence interval suggest that up to 92% of patients undergoing a surgery in a biochemically hyperthyroid state will not develop a thyroid storm. Many reasons could account for the fact that patients underwent surgery despite being in a hyperthyroid state. These reasons include anaesthesiologists and surgeon’s level of experience, patients who do not respond to pharmacological treatment, and the low overall risk of a thyroid storm.

The exact pathophysiology of thyroid storm is not well understood. One well accepted theory is that there may be a heightened response to thyroid hormone, an increased or abrupt availability of free hormones and enhanced binding to thyroid hormone receptors [1]. The management of thyroid storm is very similar to Graves’ disease. The ATDs are used at different dosage and frequency with supportive therapy [1]. The ATDs act to decrease synthesis, peripheral conversion and secretion of thyroid hormone. Thus, the fact that 46% of patients were on at least one ATD may mitigate a thyroid storm as the thyroid hormone availability is being haltered.

The literature has no established cutoffs of serum T4 or T3 that differentiates uncomplicated thyrotoxicosis from crisis [4, 8]. Many studies are leaning towards the presence of a normal serum free T3 levels with increased serum free T4 levels and suppressed TSH levels. Akamizu et al. 2012, suggested that the mean serum free T4 and free T3 concentrations were not significantly different amongst patients in a hyperthyroid state compared to patients with thyroid storm. In our sample, a comparison between uncomplicated thyrotoxicosis from crisis was not established as no patient developed a thyroid storm. However, the 95% confidence interval of TSH, free T4, free T3 and total T3 levels was calculated. Our data indicates in a hyperthyroid population, the 95% confidence interval for true free T4 level is between 18.02 and 41.15 pmol/L, for true free T3 level is 3.17 and 9.52 pmol/L and for true total T3 level is between 4.89 and 18.15 nmol/L. The 95% confidence interval for true TSH level is not statistically significant. This data can be used as a reference in the clinical practice when a hyperthyroid patient requires a surgery but the sample size is to small to establish significant cutoffs.

This study has several limitations. This retrospective study was conducted at two Canadian centers from January 2006 to December 2016. The number of patients with Graves’ disease undergoing surgery is relatively small so conclusions are difficult to ascertain. This study also reflects the work of high volume thyroid surgeons. Studies have shown improved patient outcomes to be associated with thyroidectomy volume. Thus, the results may not be generalized. Cases from Montreal were done at a tertiary center whilst the cases in Nova Scotia were done at a community center and this may as well affect the generalizability of the result. Furthermore, the data analyzed was dependent on the information available in the charts. For instance, the thyroid hormone levels obtained were the last set of blood work preceding surgery but that was variable in terms of when it was withdrawn and this time lag in some cases may have converted the patient to euthyroid state if they were still medicated. Furthermore, it must be noted that not all patient had their free T3 levels evaluated.

## Conclusion

Thyroid storm is a rare and life threatening complication associated with high mortality and as such centers are reluctant to operate on a patient in a hyperthyroid state. This study was conducted to reflect the Canadian experience with Graves’ patients in a hyperthyroid state undergoing surgery. In our study, the majority of the patients were medicated in attempt to render them euthyroid. Nonetheless, the patients that remained biochemically hyperthyroid and underwent a thyroidectomy did not develop a thyroid storm. Caution must be used when interpreting these results as the sample size is small and reflects a heterogeneous population. As a result, current practices should not be changed. Prospective studies with larger sample sizes are needed to better understand the likelihood of thyroid storm in hyperthyroid patients.

## Abbreviations

ATD: Antithyroid drugs
CI: Confidence interval
GD: Graves’ disease
RAI: Radioiodine ablation
T3: Triiodothyronine
T4: Thyroxine
TSH: Thyroid stimulating hormone
